# Anxiety and Emotional Intelligence: Comparisons Between Combat Sports, Gender and Levels Using the Trait Meta-Mood Scale and the Inventory of Situations and Anxiety Response

**DOI:** 10.3389/fpsyg.2020.00130

**Published:** 2020-02-04

**Authors:** María Merino Fernández, Ciro José Brito, Bianca Miarka, Alfonso Lopéz Díaz-de-Durana

**Affiliations:** ^1^Department of Sports, Faculty of Physical Activity and Sport Sciences-INEF, Universidad Politécnica de Madrid (UPM), Madrid, Spain; ^2^Faculty of Health Sciences, Universidad Francisco de Vitoria (UFV), Madrid, Spain; ^3^Faculty of Physical Education and Sport, Federal University of Juiz de Fora, Juiz de Fora, Brazil; ^4^Laboratory of Psychophysiology and Performance in Sports & Combats, School of Physical Education and Sport, Federal University of Rio de Janeiro, Rio de Janeiro, Brazil

**Keywords:** mood, martial arts, psychology, sports, sexual and gender disorders, anxiety

## Abstract

The present study compared emotional intelligence and anxiety between six combat sports of lower, intermediate and high-level female and male athletes. The sample was composed by 444 athletes (age: 24.7 ± 8.8 years, body mass: 72.4 ± 12.1 kg, height: 1.82 ± 0.3 m, and practice time: 13.1 ± 7.4 years) separated by sex (male *n* = 273, female *n* = 171) from different combat sports (jiu-jitsu *n* = 142, judo *n* = 137, karate *n* = 57, kendo *n* = 63, taekwondo *n* = 25, and freestyle wrestling *n* = 20) of three levels (high-level *n* = 57, intermediate *n* = 137 and low-level *n* = 142). Inventory of situations and anxiety response (ISRA) provided an independent evaluation for the three systems: cognitive, motor and physiological, as well as a total with four factors of analysis (anxiety before the evaluation, interpersonal, phobic and before habitual, and daily situations). Trait Meta-Mood Scale (TMMS-24) verified emotional intelligence scales. Descriptive results are demonstrated by percentage or median (first quartile Q1; third quartile Q3), Kruskal-Wallis and Mann-Whitney tests were conducted to compare groups, *p* ≤ 0.05. The main results demonstrated 10% more total anxiety for wrestling and judo compared to the other groups (*p* ≤ 0.05). Female athletes showed 15% more anxiety than men, while emotional attention demonstrated 10% better results for women. Significant differences were observed between high- versus low-level athletes in the total anxiety with 85 (44; 143) versus 122 (69; 186) of ISRA index and emotional repair with 30 (25; 34) versus 27 (22; 32) of TMMS-24 index. Emotional intelligence seems to be higher in female and in higher level, while anxiety appears to be prevalent in judo and wrestling, low-level and in female athletes. These outcomes provide support for the hypothesis that emotional abilities are an important contributor to emotional intelligence, particularly differentiating high level athletes than other levels. Results can be incorporated into strategies for reducing anxiety and improving emotional intelligence, considering particularities of gender and level groups.

## Introduction

Anxiety is a sensation of uneasiness and worry, typically generalized and unfocused as an overreaction to a condition that is only subjectively seen as intimidating (Brandt et al., 2018; Tahtinen and Kristjansdottir, 2018). This feeling has been an essential concept for sports psychology and has demanded intensive investigation in combat sports for its effects on championship performance in karate (Friesen et al., 2018), judo (Matsumoto et al., 2000; Interdonato et al., 2013), kendo (Usui et al., 2018), jiu-jitsu (Andreato et al., 2014), taekwondo (Maloney et al., 2018), and wrestling (Bawa, 2010). Despite such well-known concern, martial arts and combat sports practices that originally bring philosophical aspects are supposed to assist practitioners in self-control, an aspect of inhibitory control, as the ability to regulate one’s emotions, thoughts, and behavior in the face of anxieties and impulses.

One of the major difficulties confronting the researcher of combat sports in anxiety studies is the assessment of situational anxiety response or state anxiety. This assessment has been showed using behavioral (Tiric-Campara et al., 2012), physiological (Capranica et al., 2017), and/or with self-report techniques (Cerin and Barnett, 2011; Coswig et al., 2018). A variety of self-report procedures in combat sports has been used with a practical degree of success. A collective instrument to measure anxiety is the item response scale, in which the total item scores replied in the “anxious” way is the quantity of anxiety (Cheng and McCarthy, 2018). Preceding authors have developed anxiety traits questionnaires that were especially tailored to sports and was denominated as the sport competition anxiety test (SCAT) used in karate (Terry and Slade, 1995) and judo with higher values for female than male athletes (Wong et al., 2006; Interdonato et al., 2013). Recently, comparisons of mood states, using the Brunel Mood Scale (BRUMS), associated with outcomes achieved by female and male athletes in high-level judo and Brazilian jiu-jitsu (BJJ) championships demonstrated that female judo athletes had higher depression and vigor index than BJJ athletes (Brandt et al., 2019), while the logistic regression revealed that higher levels of anger and tension increased athletes’ chances of performing well in a match by 23% and 13%, respectively (Brandt et al., 2019). Overall, authors observed a significant relationship between mood state and sports performance (Brandt et al., 2019).

Presently, two different lines of study can be found in meta-mood combat sports research. The first one is focused on insightful experience of mood as state, as BRUMS (Wong et al., 2006; Brandt et al., 2019). Much of this investigation is concerned in investigating how a fighter’s thoughts may be affected by his/her mood state, and in considerate the various types of mood regulation (Jin, 1992; Terry and Slade, 1995). In addition, the second line is interested in more stable affective capacities that people routinely use to experience their feelings and moods (Salovey et al., 1995; Filaire et al., 2011; Espinoza-Venegas et al., 2015). This approach is denominated trait-meta mood research for which Salovey et al. (1995) developed the Trait Meta-Mood Scale (TMMS). In the present article we focused on this latter approach in combat sports, using a brief description of the TMMS-24, its three dimensions (Attention, Clarity, and Repair). Consequently, coaching staff and athletes should monitor athletes’ mood and anxiety states to ensure that they are in optimal condition to perform and use psychological interferences to care athlete’s preparation. However, more studies have compared gender differences, anxiety, and emotional intelligence levels between martial arts practice and combat sports.

Researchers recognize that any measure of sport anxiety must take into consideration cognitive anxiety (negative thoughts, worry) and somatic anxiety (physiological response) (Terry and Slade, 1995). The Competitive State Anxiety Inventory (CSAI-2) considers the difference between Anxiety state (i.e., momentary anxiety) and Anxiety trait (i.e., chronic anxiety) and distinguishes between cognitive and somatic anxiety (Smith et al., 2006). The CSAI-2 verified effects of anxiety in novice karate performers on visual search strategy, changing their peripheral narrowing or increasing susceptibility to peripheral distractors in response to taped offensive karate sequences (Williams and Elliott, 1999). Using CSAI-2, preceding authors demonstrated that cognitive and somatic anxiety were higher in interregional compared to regional judo championships with positive associations with cortisol (Filaire et al., 2001). Bringing together the behavioral and interactive approaches in the development of the Anxiety Situation and Response Inventory – ISRA (Miguel-Tobal and Cano-Vindel, 2002), an Inventory in the Situation-Response format, designed to assess the frequency with which manifests itself in a series of cognitive responses, physiological and motor disorders before different situations (i.e., of evaluation, interpersonal, phobic, and of the daily life). The ISRA psychometric properties, concerning convergent validity, as well as their capacity for discrimination, both between groups with various anxiety disorders, as between anxiety and depression (Miguel-Tobal and Cano-Vindel, 2002). Several adaptations of the original inventory have also been elaborated, oriented toward the valuation of anxiety in highly specific contexts, such as anxiety for medical revisions and to value the eagerness of the pilots of the Air Force (Miguel-Tobal and Cano-Vindel, 2002). The practical applications of these psychological instruments in combat sports are important to incorporate self-regulation strategies.

Self-regulation of anxiety plays an essential role in competitive and professional combat sport (Diamond, 2012; Breitschuh et al., 2018). During championship, for example, it is important to stick to one’s competition plan and not give up when it gets tough but rather to mobilize additional energy (Alsamir Tibana et al., 2019). Self-regulation of anxiety is similarly a required condition for maintaining practice during long and exhausting periods of training that require concentration (Finkenberg et al., 1992; Lakes et al., 2013). This is particularly crucial if the training is under conditions of high demand and, in itself is not very motivating, but nevertheless active (Jin, 1992; Matsumoto et al., 2000). The contributions of preceding studies have shown anxiety as an eminent problem in different combat sports (Terry and Slade, 1995; Williams and Elliott, 1999; Tiric-Campara et al., 2012; Interdonato et al., 2013). However, the actual psychological distance between points or scores on these scales is typically unknown, while the Interval Scale of Anxiety Response Scale (ISAR) can provide a sensitive instrument for measuring situational anxiety (Heaton et al., 2007).

Therefore, the aim was to compare emotional intelligence and anxiety between six combat sports of lower, intermediate and high-level female and male athletes. Based on these assumptions, our first hypothesis suggested that those athletes who perform combat sports and have a higher performance would show higher emotional intelligence and lower anxiety levels, measured by the ISRA than athletes of combat sports with a lower performance. The second hypothesis is regarding gender differences, being that female who train and compete in combat sports will have higher anxiety scores than male, as measured through the ISRA.

## Materials and Methods

### Study Design

Present study is a cross-sectional study. We applied a descriptive method in which anxiety and emotional intelligence in athletes were analyzed according to their competitive levels. As dependent variables we used: emotional intelligence and anxiety; and as independent variables: competitive levels (low, medium and high) and gender (male and female). The measures were carried out in two situations: (a) competitions when the athletes were in qualification procedures and weighing (24-h before the competition); and (b) during national trainings. All measurements were performed by a single researcher, with ISRA applied first, followed by the TMMS-24. The aims and risks of the study were informed to the participants and they signed the Informed Consent Form. This protocol was approved by the Research Ethics Committee of the University in which it was performed.

### Sample

The following inclusion criteria were applied: (a) be ≥15 years of age, (b) to practice and compete in jiu-jitsu, judo, karate, kendo, taekwondo and wrestling (≥5 years). 1,400 questionnaires were applied to carry out the study, of which 922 were returned, and 476 were rejected because they presented incomplete information, therefore the final sample was composed by 444 athletes (age: 24.7 ± 8.8 years, body mass: 72.4 ± 12.1 kg, height: 1.82 ± 0.3 m, and practice time: 13.1 ± 7.4 years) separated by sex (female = 171, male = 273) from different combat sports (Jiu-jitsu = 142, judo = 137, karate = 57, kendo = 63, taekwondo = 25, and freestyle wrestling = 20) of three levels (high level = 57, intermediate = 137, and lower level = 142). Regarding performance, the athletes were classified as: (a) low (compete, but without regional medals; female = 59 and male = 126); (b) medium (compete and own regional and national medals; female = 80 and male = 112); and (c) high (they compete and were classified in the top 5 in the continental or world championships; female = 33 and male = 36).

### Measurements

The Trait Meta-Mood Scale (TMMS-24) (Salovey et al., 1995) or Emotional Intelligence Scale translated into Spanish (Fernández-Berrocal et al., 2004) was used. This inventory consists of 24 items that are subdivided into three subscales or dimensions: (a) emotional attention; (b) emotional clarity; and (c) emotional repair. The score for each of these subscales is classified into three ranges. For the emotional perceived subscale, the middle score range (22–32 in male; 25–35 in female) indicates adequate emotional attention, and scores in the high (>33 in male; >36 in female) or low (<21 in male; <24 in female) range indicate that emotional attention should be improved. In contrast, for the clarity subscale, scores in the low range indicate a need for improvement (<25 in male, <23 in female), those in the middle range (26–35 in male; 24–34 in female) indicate adequate clarity, and those in the high range (>36 in male; >35 in female) indicate excellent emotional clarity. Likewise, in the emotional repair subscale, low scores (<23 in male and female) indicate the need for improvement, scores in the middle range (24–35 in male, and 24–34 in female) indicate adequate repair, and high scores (>36 in male, >35 in female) indicate excellent emotional repair. In the questionnaire, individuals must rate each of their responses on a Likert scale from one to five points to indicate their level of agreement. The total score is obtained by adding the responses from each sub-scale, each of which ranges from eight to 40 points.

#### ISRA

Inventory of situations and anxiety responses provides an independent evaluation for the three response systems: cognitive, motor and physiological, as well as a total. It also includes four factors of analysis: anxiety before the evaluation (FI), interpersonal (F-II), phobic (F-III), and before habitual and daily situations (FIV). This enabled us to develop a profile of individual reactivity and measurements followed preceding protocol (Miguel-Tobal and Cano-Vindel, 2002). The athlete had to indicate the frequency with which each one of the anxiety responses appears in the proposed situation according to a Likert scale of 5 points. The original version of the ISRA was used consisting of 224 items in an open situation. These items are composed of an interaction of 22 situations and 24 responses. We obtained direct scores and subsequently calculated a percentile that offers a scale for each subject in each of the measures. The objective of passing this inventory was to see how the anxiety of the subjects was distributed in terms of the triple response system (motor, physiological, and cognitive) and to observe their general anxiety. Regarding the factors that it offers us, we consider the FI to be important since it measures the anxiety before the evaluation. In any case, the others were analyzed to see if there were any other differences. In the case of athletes, one of the factors that is indicated as causing anxiety is precisely the fear of being evaluated or doing it badly and failing (factor I of the ISRA measures the anxiety before the evaluation).

To estimate the internal reliability coefficient of the questionnaire items, the reliability used the Cronbach’s alpha (a) coefficient, since an analysis based on internal consistency of the items (Artioli et al., 2010; Molanorouzi et al., 2014). The coefficient “alpha” is the designation of the statistical procedure using scales with Likert type items and this method was used because the responses to the items are distributed by an ordinal scale (Shojima and Toyoda, 2002; Okada, 2015). The methods based on the internal consistency of the items tend to supplant the stability-based coefficients, thus requiring higher than or equal to 0.70 (Moret et al., 1993; Sun et al., 2007). The reliability of the TMMS-24 instrument for the Spanish subscales were Emotional Attention α = 0.90, Emotional Clarity α = 0.90, and Emotional Repair α = 0.86 (Fernández-Berrocal et al., 2004). In addition, the spanish version of the Inventory of Situations and Responses of Anxiety (ISRA) (Miguel-Tobal and Cano-Vindel, 2002). The reliability of the TMMS-24 instrument for the Spanish subscales were α = 0.87 cognitive, α = 0.85 physiological, and α = 0.74 motor responses of anxiety (Martínez-Sánchez et al., 1995; Ziv and Lidor, 2013).

### Statistical Analysis

Descriptive data is presented as median [25th percentile; 75th percentile] values and the Kruskal-Wallis One-way Analysis of Variance and pairwise Mann-Whitney tests were conducted to compare ISRA frequencies between groups. For the Kruskal-Wallis, the effect size was calculated as ES = √(chi^2^/N), where chi^2^ is derived from the Kruskal-Wallis test results and N is the total number of observations (Rosenthal and DiMatteo, 2001). For the Mann-Whitney test, the effect size was calculated as *r* = Z/√N, where Z is derived from the Mann-Whitney test results and N is the total number of observations, and ES was interpreted as follows: small (*r* = 0.1), medium (*r* = 0.3), or large (*r* = 0.5). The significance level of *p* ≤ 0.05 was used. All analyses were conducted using SPSS 20.0 for Windows.

## Results

Descriptive analysis of ISRA and TMMS-24 between combat sports are shown in Table 1. Jiu-jitsu athletes demonstrated lower values of cognitive anxiety than judo (*p* = 0.26) or kendo athletes (*p* = 0.033). The Judo group showed higher cognitive anxiety than karate (*p* = 0.005), and karate presented lower levels of cognitive anxiety than kendo (*p* = 0.006). Kendo was different from taekwondo (*p* = 0.047). Judo athletes presented higher ISRA T than Jiu-jitsu (*p* = 0.040) or kendo athletes (*p* = 0.005), while karate presented lower values of total anxiety than kendo (*p* = 0.021) and wrestling (*p* = 0.038).

**TABLE 1:** Descriptive and inferential analysis of combat sports anxiety and emotional intelligence comparisons (median, first, and third quartiles).

Factors	Jiu-jitsu	Judo	Karate	Kendo	Taekwondo	Wrestling	Statistical Inference			
	μ (25th; 75th)	μ (25th; 75th)	μ (25th; 75th)	μ (25th; 75th)	μ (25th; 75th)	μ (25th; 75th)	X^2^	df	*P*	ES
ISRA C	55 (30; 70)	62 (35; 89)	44.0 (25.5; 68.5)	59 (38; 93)	66 (31; 80.5)	57.5 (35.5; 65.8)	134.245	5	0.04	0.17
ISRA M	24 (11; 43)	30 (12; 63)	20 (9.5; 39.5)	28 (10; 49)	22 (10.5; 40.5)	47 (27; 55)	10875	5	0.09	0.16
ISRA F	27 (12; 50)	29 (13; 58)	21 (9.5; 40)	24 (12; 48)	32 (11.5; 58.5)	39.5 (12.8; 55)	56.901	5	0.46	0.11
ISRA T	108 (56; 161)	125 (60; 214)	85 (45.5; 147)	113 (73; 181)	107 (56.5: 190.5)	153.5 (92.5; 171)	113.352	5	0.08	0.16
I F1	47 (25; 74)	60 (28; 88)	51 (22.5; 70)	48 (32; 78)	49 (28.5; 79.5)	56.5 (33.3; 77)	6.183	5	0.42	0.12
I F2	10 (4; 19)	13 (5; 27)	8 (4.5; 14.5)	10 (6; 21)	9 (3; 30.5)	18.5 (4.8; 22)	1167	5	0.09	0.16
I F3	14 (4; 30)	22 (6; 43)	13 (3; 30.5)	14 (4; 33)	13 (5.5; 31)	32 (23.5; 40.8)	115.400	5	0.07	0.16
I F4	5 (2; 16)	9 (3; 20)	6 (2.5; 13.5)	7 (3; 18)	7 (2.5; 14.5)	13.5 (3.3; 29)	8.600	5	0.23	0.13
IE 1	23 (19; 27)	23 (19; 28.5)	22 (18; 27)	21 (17; 29)	22 (18; 26)	21.5 (15.3; 25.8)	536	5	0.53	0.06
IE 2	28 (23; 32)	26 (22; 31)	30 (23; 32)	28 (23; 32)	31 (24; 33)	29.5 (20; 33)	6.284	5	0.39	0.07
IE 3	29 (25; 32)	28 (24; 33)	30 (25; 33)	26 (25; 32)	27 (23.5; 33)	28.5 (18.3; 36.3)	2.164	5	0.90	0.06

*ISRA C, cognitive anxiety; ISRA M, motor anxiety; ISRA F, physiological anxiety; ISRA T, total anxiety; I F1, anxiety before the evaluation; I F2, interpersonal anxiety; IF 3, phobic anxiety; I F4, anxiety before habitual and daily situation; IE 1, emotional attention; IE 2, clarity; IE3, emotional repair; μ, median; 25th, 1st quartile; 75th, 3rd quartile; X^2^, Chi-square; df, degrees of freedom; *P*, *P* calculated; ES, effect size.*

The Jiu-jitsu group showed similar results of total anxiety from karate (*p* = 0.17), kendo (*p* = 0.21), taekwondo (*p* = 0.65), and wrestling (*p* = 0.19). Judo presented similar results of total anxiety when compared with kendo (*p* = 0.57), taekwondo (*p* = 0.38), and wrestling (*p* = 0.87). Karate presented similar results for total anxiety as taekwondo (*p* = 0.19). Total anxiety comparison for kendo, taekwondo and wrestling did not present a significant effect (*p* = 0.532 and *p* = 0.85). Similar results of total anxiety were observed between jiu-jitsu (*p* = 0.52), karate (*p* = 0.20), taekwondo (*p* = 0.37) and wrestling (*p* = 0.87); judo and kendo (*p* = 0.75), taekwondo (*p* = 0.49), and wrestling (*p* = 0.28).

Judo had higher motor anxiety than karate (*p* = 0.015) and Jiu-jitsu (*p* = 0.047), and karate had lower motor anxiety than wrestling (*p* = 0.015). Jiu-jitsu had similar motor anxiety to karate (*p* = 0.32), kendo (*p* = 0.42), taekwondo (*p* = 0.85), and wrestling (*p* = 0.069); karate was similar to kendo (*p* = 0.13) and taekwondo (*p* = 0.60), while kendo was similar to taekwondo (*p* = 0.44) and wrestling (*p* = 0.14); in addition, taekwondo and wrestling were similar (*p* = 0.056).

Judo demonstrated higher interpersonal anxiety than jiu-jitsu (*p* = 0.016) and karate (*p* = 0.007). Kendo showed higher values of interpersonal anxiety than karate (*p* = 0.038). Judo and wrestling presented a higher result of phobic anxiety than jiu-jitsu (*p* = 0.02 and *p* = 0.019). Jiu-jitsu presented similar results to karate (*p* = 0.35), kendo (*p* = 0.2), taekwondo (*p* = 0.69), and wrestling (*p* = 0.20). Judo had similar results of interpersonal anxiety when compared to kendo (*p* = 0.32), taekwondo (*p* = 0.32), and wrestling (*p* = 0.91). Karate demonstrated similar results for interpersonal anxiety when compared with taekwondo (*p* = 0.44) and wrestling (*p* = 0.65), and taekwondo had similar results of interpersonal anxiety as wrestling (*p* = 0.7). Wrestling presented higher phobic anxiety than karate (*p* = 0.026) and taekwondo (*p* = 0.03).

Jiu-jitsu presented similar result of phobic anxiety when compared with karate (*p* = 0.88), kendo (*p* = 0.52), and taekwondo (*p* = 0.95). Judo presented similar results for phobic anxiety to karate (*p* = 0.056), kendo (*p* = 0.19), taekwondo (*p* = 0.15), and wrestling (*p* = 0.34). Karate and taekwondo presented similar results (*p* = 0.98). No effects in TMMS-24 comparisons were observed between combat sports when compared to emotional attention (*p* = 0.54), clarity (*p* = 0.39) and emotional repair (*p* = 0.90). A descriptive analysis of ISRA and TMMS-24 between levels are in Table 2.

**TABLE 2:** Descriptive and inferential analysis of different levels anxiety and emotional intelligence comparisons (median, first, and third quartiles).

Factors	High level	Intermediate level	Low level	Statistical inferences			
	μ (25th; 75th)	μ (25th; 75th)	μ (25th; 75th)	X^2^	df	*P*	ES
ISRA C	41 (22; 65.5)	54 (33; 80)	62 (35; 87)	11.524	2	0.003	0.16
ISRA M	21 (9.5; 30)	27 (10; 52)	28 (13; 53)	7.183	2	0.028	0.13
ISRA F	21 (10; 36)	26 (12; 51)	30 (13.5; 54.5)	6.225	2	0.044	0.12
ISRA T	85 (44.5; 143)	116 (60; 179)	122 (69; 186.5)	11.101	2	0.004	0.16
I F1	37 (24; 63)	52 (30; 79)	56 (27; 82.5)	7.448	2	0.024	0.13
I F2	6 (3; 15.5)	11 (5; 22)	12 (5; 25)	14.326	2	0.001	0.18
I F3	12 (3; 29.5)	15 (4; 35)	20 (6; 41)	7.531	2	0.023	0.13
I F4	5 (1; 12.5)	6 (3; 17)	8 (3; 17)	4.588	2	0.101	0.10
IE 1	22 (17; 27)	14 (19; 28)	22 (17.5; 27)	5.390	2	0.68	0.01
IE 2	30 (25; 32)	27 (23; 32)	27 (22; 32)	7.444	2	0.024	0.12
IE 3	30 (25; 34.5)	30 (25; 33)	27 (22; 32)	12.794	2	0.003	0.14

*ISRA C, cognitive anxiety; ISRA M, motor anxiety; ISRA F, physiological anxiety; ISRA T, total anxiety; I F1, anxiety before the evaluation; I F2, interpersonal anxiety; IF 3, phobic anxiety; I F4, anxiety before habitual and daily situation; IE 1, emotional attention; IE 2, clarity; IE3, emotional repair; μ, median; 25th, 1st quartile; 75th, 3rd quartile; X^2^, Chi-square; df, degrees of freedom; *P*, *P* calculated; ES, effect size.*

High level athletes presented lower values than lower level athletes in cognitive anxiety (*p* = 0.002), motor anxiety (*p* = 0.028), physiological anxiety (*p* = 0.039), total anxiety (*p* = 0.003), before the evaluation (*p* = 0.024), interpersonal anxiety (*p* = 0.01) and before everyday situations (*p* = 0.031). Regarding the interaction between level and combat sport groups, a main effect was observed in karate athletes (X^2^ = 6.468, df = 2, *p* = 0.039), high level karate group had lower motor anxiety than the intermediate athletes (*p* = 0.032).

In addition, high level athletes presented lower values than intermediate athletes for total anxiety (*p* = 0.028), before the evaluation (*p* = 0.003) and phobic anxiety (*p* = 0.011). Concerning the interaction between level and combat sports, a main effect was observed in phobic anxiety of karate athletes (X^2^ = 6.193, df = 2, *p* = 0.045), where high level group demonstrated lower phobic anxiety values than the intermediate athletes (*p* = 0.045). Significant differences in TMMS-24 comparisons between levels were observed in clarity (X^2^ = 7.444, df = 2, *p* = 0.024) with higher scores for the high level group versus the low level group (*p* = 0.013), and in emotional repair (X^2^ = 12.794, df = 2, *p* = 0.002) with lower scores to the low level group when compared with the intermediate group (*p* = 0.003) and high level group (*p* = 0.002). Regarding the interaction between level and combat sport groups, significant differences were observed in emotional repair (X^2^ = 8.943, df = 2, *p* = 0.011) jiu-jitsu athletes presented lower values in lower level group than the intermediate athletes (*p* = 0.01), kendo also demonstrated differences between levels in emotional repair (X^2^ = 7.362, df = 2, *p* = 0.025) with higher emotional repair on high level group compared with the lower level athletes (*p* = 0.025). Karate athletes demonstrated significant effects in emotional attention when compared level groups (X^2^ = 6.537, df = 2, *p* = 0.038), high level athletes presented higher emotional attention than the lower level group (*p* = 0.034). No effects in emotional attention (*p* = 0.068) were observed. Descriptive analysis of ISRA and TMMS-24 between male and female athletes are in Table 3.

**TABLE 3:** Descriptive and inferential analysis of gender anxiety and emotional intelligence comparisons (median, first, and third quartiles).

Factors	Female	Male	Statistical inferences			
	μ (25th; 75th)	μ (25th; 75th)	*U*	*Z*	*P*	ES
ISRA C	68 (43; 88)	46 (27; 71)	16833	4.997	0.001	0.24
ISRA M	35 (20; 55)	20 (8; 45.3)	16963	4.899	0.001	0.23
ISRA F	34 (20; 55)	20 (10; 45)	17597	4.419	0.001	0.21
ISRA T	138 (95; 194)	90 (48; 158.5)	16587	5.183	0.001	0.25
I F1	63 (38; 85)	42.5 (22.8; 68)	15932	5.680	0.001	0.27
I F2	10 (6; 25)	10 (4; 21)	21075	1.784	0.07	0.08
I F3	25 (6; 41)	13 (4; 31)	19109	3.274	0.001	0.16
I F4	10 (4; 18)	5 (1; 15)	1833.5	3.869	0.001	0.18
IE 1	24 (20; 28)	21 (17; 27)	1941.5	3.417	0.001	0.16
IE 2	27 (23; 32)	28 (23; 32)	23.646	1.323.2	0.62	0.06
IE 3	29 (24; 33.8)	29 (24; 33)	22.811	1.323.4	0.57	0.06

*ISRA C, cognitive anxiety; ISRA M, motor anxiety; ISRA F, physiological anxiety; ISRA T, total anxiety; I F1, anxiety before the evaluation; I F2, interpersonal anxiety; IF 3, phobic anxiety; I F4, anxiety before habitual and daily situation; IE 1, emotional attention; IE 2, clarity; IE3, emotional repair; μ, median; 25th, 1st quartile; 75th, 3rd quartile; X^2^, Chi-square; df, degrees of freedom; *P*, *P* calculated; ES, effect size.*

Female athletes presented higher cognitive, motor, physiological and total anxiety than male athletes (*p* = 0.001). Significant differences in TMMS-24 comparisons between genders were observed in clarity, with higher values for female compared with male (*p* < 0.001). When observed the interaction between sex and level groups in TMMS-24, comparisons indicated differences in the clarity dimension (X^2^ = 6.073, df = 2, *p* = 0.048) with higher values by female and male high-level than female low-level athletes (*p* = 0.044). No effects were observed in clarity (*p* = 0.95) and emotional repair (*p* = 0.57) between male and female combat athletes.

## Discussion

This study examined the associations between important factors in the field of sports psychology (i.e., anxiety and emotional intelligence), comparing emotional intelligence and anxiety between six combat sports of lower, intermediate and high-level female and male athletes. The main results demonstrated higher values of cognitive and motor anxieties in judo, taekwondo and kendo than for jiu-jitsu, wrestling and karate groups. Judo athletes demonstrated higher phobic and interpersonal anxieties than the other groups. No difference in emotional intelligence was observed between modalities, which may suggest that anxiety is more associated with other factors such as gender and competitive level. Our data showed an inverse relationship between anxiety levels and expertise in combat sports practices, while females revealed higher anxiety than male athletes for all the analyzed variables. On the other hand, females demonstrated higher scores in emotional attention than male athletes, but both groups were classified as having low emotional attention, while high-level athletes showed better (classified as adequate) clarity and emotional repair than low-level athletes. Self-knowledge about the emotions, feelings, and moods together with the skill domain can help an athlete improve their performance (Cerin and Barnett, 2011; Tiric-Campara et al., 2012).

Emotional intelligence of applied psychology suggested the existence of three major conceptual models: (i) a transverse section of interrelated emotional and social competencies, abilities, and facilitator that influence intelligent behavior, called emotional clarity; (ii) a wide range of competencies and abilities that increase work performance, called emotional attention; and (iii) the ability to perceive, understand, manage, and regulate one’s emotions, as well as the emotions of others, called emotional repair (Espinoza-Venegas et al., 2015). Despite the importance of emotional intelligence in combat sports, few studies have been conducted with this theme (Filaire et al., 2011).

Recently, in a randomized controlled trial, the effects of karate versus a mind-based stress reduction intervention on well-being and cognitive functioning in older adults demonstrated that both methods showed only small training effects concerning the assessed emotional and cognitive parameters (Jansen et al., 2017). This emotional self-control associated with anxiety levels has not been studied in different combat sports practices and in adults who are competitors in different levels until now. Assuming that combat sports present particular philosophies empirically associated with emotional control, it is significant to verify associations between expertise and controlled levels of anxiety.

The symptoms of anxiety can be recognized on different levels (Cheng and McCarthy, 2018), such as Cognitive (i.e., by a thought process), Somatic (i.e., by a physical response) and Behavioral (i.e., by patterns of behavior). For instance, a preceding report found that winning male college taekwondo athletes showed higher self-confidence and lower cognitive and somatic anxiety than their losing counterparts (Chapman et al., 1997). Although different studies have already verified the state and trait anxiety in combat sports, no studies have compared the symptoms of anxiety and emotional intelligence scale in different combat modalities.

Our gender comparisons shown larger effect sizes of anxiety variables and significant differences; female athletes had ∼35% more total anxiety than males who had ∼30% less cognitive anxiety characterized by indecision, sense of confusion, feeling heavy, negative thoughts, poor concentration, irritability, fear, forgetfulness, loss of confidence, and other negative factors. Furthermore, female presented ∼60% more physiological and ∼60% more motor anxieties. Female participants presented common indications of physiological anxiety such as increased blood pressure, respiration rate, sweating, adrenaline surge, needing to urinate, muscular tension, trembling, incessant talking and sleeplessness. In addition, motor anxiety was characterized by lethargic movements, inhibited posture and uncontrolled motor feeling. Females also had high scores of interpersonal and phobic anxieties which could be associated with presented symptoms. This outcome is consistent with an earlier study that suggested significant differences between male and female athletes during a judo championship (Interdonato et al., 2013).

To the author’s knowledge, TMMS-24 was measured here for the first time in combat sports, with gender differences in emotional attention set shifting ability. Female athletes with higher total anxiety and worrisome thoughts did not associate difficulty in anxiety with emotional attention capacity; on the contrary, these results suggest that activation caused by anxiety may increase emotional attention. Although this study demonstrated that female athletes were related to anxiety and worry, it remains unclear whether female anxieties are related to a generalized mechanism of emotion repair. Regarding emotional intelligence differences between the level groups, TMMS-24 for the high-level group demonstrated ∼40% less total anxiety and ∼10% more clarity and repair than the low-level group. This result demonstrated that emotional intelligence and expertise are associated in combat sports, as well as the anxiety reduction, especially cognitive anxiety, which is associated with negative thoughts, poor concentration, fear and loss of confidence. Coco et al. (2019) indicated that judo turns out to be a sport discipline useful to male athletes control their aggressiveness and to try of overcoming their limits. Another study recently showed a positive association between religious beliefs with anxiety in competitive judo athletes (Moghadam et al., 2015).

Although such settings have greater external validity, the variables of interest are more difficult to control and measure than in a laboratory environment. Present study indicated as a limitation the fact that information collected was a cross-sectionally design, occurring during an over short period of time, it did not show a long-term profile of combat athletes. No effects of age, body mass, height or practice time were observed in present research. However, preceding reports indicated that a rapid weight loss is associated with high anger, confusion and greater total mood disturbance at the official weigh-in in Mixed Martial Arts athletes (Brandt et al., 2018). Recently, findings indicated the association between obesity and hyperactivity and anxiety among female school students in Japan (Suzuki et al., 2019). Future studies would do longitudinal studies, conducting several observations of the same athletes over a period of time in combat sports, verifying the relationship between anxiety, emotional intelligence, and anthropometric factors.

The benefit of a longitudinal study is that researchers would be able to detect developments or changes in the anxiety and emotional intelligence characteristics of the combat sports over a year or during sequential championships, principally comparing levels of anxiety. Our study ensured that participants were of the same age range; even so, it is important to point out a limitation for non-comparisons between combat outcomes during championships. Preceding research indicated that better performance during judo championships was associated with significantly lower levels of cognitive anxiety or higher levels of confidence (Filaire et al., 2001). Chiodo et al. (2011) also demonstrated significantly higher post-match scores emerged for anger-hostility and depression-dejection, whereas the reverse was observed for vigor-activity. In particular, results discriminated observed parameters between different competition levels of each single combat sport and highlighted significant findings in jiu-jitsu, judo, karate and kendo. This approach clarified the findings of present study, as preceding reports indicated that different attention could emerge in the same combat sport group, because taekwondo athletes who train with different approaches had different session-RPE quantifying loads (Lupo et al., 2017).

Our results indicated higher values of cognitive and motor anxieties in judo, taekwondo and kendo than in other groups. These findings highlight that judo athletes demonstrated higher phobic and interpersonal anxieties than other modalities. Recent judo rules changes have reduced male combat time to 4 minutes and moments of low-intensity combat time during the approach and gripping time before the attack (Miarka et al., 2016; Brito et al., 2017). A recent study has shown blood lactate rates associated with a protective role against fatigue toward frontal cortex and defined worse performances in backward memory capacity by conditioning strategic ability of the athletes (Coco et al., 2019). Recent study in rodents compellingly supports the knowledge that the projection of neurons extending from the CA1 region of the hippocampus and from the subiculum to the prefrontal cortex, denoted to the H-PFC pathway, that is critically involved in aspects of cognition associated to the executive function, as to the repair (Godsil et al., 2013). Simultaneously, it is becoming evident that judo fighter suffering from depression, and post-traumatic stress could display structural anomalies and unusual functional coupling within the hippocampal–prefrontal circuit (Godsil et al., 2013). This overlap might similarly be intertwined with the pathway’s evident susceptibility to stress and with its relationship to the amygdala, associated with anxiety and mood disorders (Kirkbride et al., 2012) in judo/Brazilian jiu-jitsu (Brandt et al., 2019) and MMA athletes (Brandt et al., 2018). In consequence, the H-PFC pathway could be a potentially crucial element of the anxiety in judo athletes.

Present study did not observed effects of interventions able to reduce anxiety levels and it could offer a specific target for therapeutic intervention in future studies. Furthermore, it has been shown that anxiety and stress responses to stressful motivations take account of coping strategies (Ursin, 1988). Coping depends on a positive response outcome expectancy, which again depends on a high emotional intelligence; our results of emotional clarity and repair were classified as adequate, while emotional attention had a low score. Filaire et al. (2001) indicated two styles of coping strategies that can be used when confronting an adverse event, being emotion-focused and problem-focused strategies, which could affect performance (Scanlan et al., 1991). The present findings help to create new strategies that can not only reduce the effects of anxiety, but also increase emotional intelligence, especially emotional attention, as well as creating strategies to reduce anxieties, particularly in judo, taekwondo and kendo. These modalities have meditation, as well as practicing forms that could be used as coping strategies for anxiety reduction and stress, especially in beginners and low-level competitive athletes.

Future studies could incorporate other measures, considering the limitations of the present study, as the answers that it gathers in the cognitive, physiological and motor systems allow only to obtain a general level of information, and little differentiated, of the existence of anxiety, since the sampling of responses that it contemplates is reduced; Practical applications could use our data, trying also to assess differential profiles of situational reactivity, since it does not take into account the continuous process of subject-situation-response interaction, and; in the clinical setting, to adapt the treatment to the specificity of the gender profiles of the specific combat sport and level that elicits emotional responses.

In fact, this study proposed a unique approach to characterizing the anxiety and emotional intelligence in gender, level, and combat sports. The presented results demonstrated higher values of cognitive and motor anxieties in judo, taekwondo and kendo, while judo athletes revealed higher phobic and interpersonal anxieties than other modalities. The findings indicated an inverse relationship between anxiety levels and expertise in combat sports, while females revealed higher anxiety than male athletes in all analyzed variables. Female athletes revealed higher emotional attention than male athletes, but both groups were classified with low emotional attention, while high-level athletes showed better and adequate clarity and emotional repair than the low-level athletes. These findings can be used in combination with the extensive knowledge of sport physiology as a means to support psychological preparation for combat sports, considering particularities of gender and level groups.

## Data Availability Statement

The datasets generated for this study are available on request to the corresponding author.

## Ethics Statement

Ethical review and approval was not required for the study on human participants in accordance with the local legislation and institutional requirements. The patients/participants provided their written informed consent to participate in this study.

## Author Contributions

MF and AD conceived, planned, and carried out the study. CB and BM realized statistical analysis and wrote the manuscript with input from all authors.

## Conflict of Interest

The authors declare that the research was conducted in the absence of any commercial or financial relationships that could be construed as a potential conflict of interest.
