# Corrigendum: Anxiety and Emotional Intelligence: Comparisons Between Combat Sports, Gender and Levels Using the Trait Meta-Mood Scale and the Inventory of Situations and Anxiety Response

**DOI:** 10.3389/fpsyg.2020.00889

**Published:** 2020-05-05

**Authors:** María Merino Fernández, Ciro José Brito, Bianca Miarka, Alfonso Lopéz Díaz-de-Durana

**Affiliations:** ^1^Department of Sports, Faculty of Physical Activity and Sport Sciences-INEF, Universidad Politécnica de Madrid (UPM), Madrid, Spain; ^2^Faculty of Health Sciences, Universidad Francisco de Vitoria (UFV), Madrid, Spain; ^3^Faculty of Physical Education and Sport, Federal University of Juiz de Fora, Juiz de Fora, Brazil; ^4^Laboratory of Psychophysiology and Performance in Sports & Combats, School of Physical Education and Sport, Federal University of Rio de Janeiro, Rio de Janeiro, Brazil

**Keywords:** mood, martial arts, psychology, sports, sexual and gender disorders, anxiety

In the published article, there were errors in affiliations 1 and 2. Instead of:

^1^ Department of Sports, Faculty of Physical Activity and Sports, Technical University of Madrid (UPM), Madrid, Spain
^2^ Faculty of Education and Humanities, Francisco de Vitoria University, Madrid, Spain
it should be:
^1^ Department of Sports, Faculty of Physical Activity and Sport Sciences-INEF, Universidad Politécnica de Madrid (UPM), Madrid, Spain
^2^ Faculty of Health Sciences, Universidad Francisco de Vitoria (UFV), Madrid, Spain

The authors apologize for this error and state that this does not change the scientific conclusions of the article in any way. The original article has been updated.

